# Postbiotic Gamma-Aminobutyric Acid and Camel Milk Intervention as Innovative Trends Against Hyperglycemia and Hyperlipidemia in Streptozotocin-Induced C_57_BL/6J Diabetic Mice

**DOI:** 10.3389/fmicb.2022.943930

**Published:** 2022-07-11

**Authors:** Amro Abdelazez, Garsa Alshehry, Eman Algarni, Huda Al Jumayi, Heba Abdel-Motaal, Xiang-Chen Meng

**Affiliations:** ^1^Key Laboratory of Dairy Science, Ministry of Education, College of Food Sciences, Northeast Agricultural University, Harbin, China; ^2^Faculty of Agriculture and Forestry, Institute of Microbe and Host Health, Linyi University, Linyi, China; ^3^Department of Dairy Microbiology, Agriculture Research Centre, Animal Production Research Institute, Giza, Egypt; ^4^Department of Food Science and Nutrition, College of Sciences, Taif University, Taif, Saudi Arabia; ^5^Department of Microbiology, Agriculture Research Center, Soils, Water, Environment and Microbiology Research Institute, Giza, Egypt

**Keywords:** postbiotic, gamma-aminobutyric acid, *Lactobacillus brevis*, diabetes type 1, camel milk, C_57_BL/6 mice

## Abstract

Diabetes is a serious disease that threatens human health worldwide. The study hypothesis is to investigate the novel trends that may aid in the prevention of diabetic complications. Camel milk was presented as traditional functional food, and *Lactobacillus brevis* KLDS_1.0727_ and KLDS_1.0373_ strains were shown to synthesize postbiotic Gamma-aminobutyric acid as a potential food additive, which can therapeutically intervene against hyperglycemia and hyperlipidemia in streptozotocin-induced C_57_BL/6J mice. During a four-week timeframe, body weight and postprandial blood glucose levels were monitored. Post-euthanasia, blood plasma was obtained to investigate hyperlipidemia, insulin concentrations, liver, and renal functions. The liver, pancreas, kidney, and spleen underwent histopathological examinations. The results demonstrated that KLDS_1.0727_ and KLDS_1.0373_ (*LAC_S1_*, *LAC_S2_*) and camel milk treatments all had a significant influence on hypoglycemic activity, as evidenced by reduced postprandial blood glucose levels. *LAC_S1_*, *LAC_S2_*, and camel milk therapy significantly reduced blood hypolipidemic, and some liver enzymes such as (alanine aminotransferase and aspartate transaminase) levels. Therefore, we recommend consuming camel milk regularly and expanding its use with fermented foods containing *L. brevis*, one of the probiotics capable of producing gamma-aminobutyric acid (GABA) as future food additives that can improve human health and reduce the prevalence of several diseases disorders.

## Introduction

Diabetes is rapidly becoming a global epidemic. It is also classified as a lifestyle disease and a critical metabolic disorder that developed with postprandial hyperglycemia which is mostly caused by insulin resistance (Patil et al., 2015). Organ failure, hypertension, hyperlipidemia, cardiovascular disease, as well as pancreatic oxidative stress, all lead to *β*-cell destruction and reduced insulin sensitivity. Therefore, all of these inevitable factors may lead to mortality for diabetic patients (Di Martino et al., 2021).

Currently, scientists are looking for innovative ways to manage diabetes complications, particularly from natural sources such as functional foods. Non-pharmacological daily activities (e.g., diet, exercise, and weight loss) and pharmaceutical alternatives (e.g., insulin stimulators, insulin inhibitors, and glucosidase inhibitors) are two strategies for lowering diabetes prevalence. However, Complementary and alternative medications may have some negative side effects (Verhaegen and Van Gaal, 2021).

The interaction between microbiota and human health disorders has become increasingly emerging (Suzzi and Corsetti, 2020). The gut–brain axis provides new insight into the interactions between the intestine and the brain across different pathways and molecules, such as the intestinal nervous system, the vagus nerve, microbial metabolites, and the immune system *via* gut microbiota functions that regulate the central nervous system (Jones et al., 2020).

Before using the word “Postbiotic” or using it as a food component, the United States Food and Drug Administration has required that it either go through pre-market clearance as an additive or be examined by experts to establish if it is generally recognized as safe (Salminen et al., 2021). Several investigations have demonstrated postbiotics as bioactive molecules produced by microorganisms’ metabolism and released into the microbial environment before or after they die. Enzymes, polysaccharides, organic acids, short-chain fatty acids, cell surface proteins, vitamins, and lipids are examples of these essential substances (Aguilar-Toalá et al., 2018).

Gamma-aminobutyric acid (GABA) has potential bio-vital postbiotic functions and is authorized as a dietary supplement with growing evidence of its influence on the gut–brain axis and systemic metabolic health (Mancini et al., 2019). Additionally, it has been shown to exhibit antihypertensive and antidepressive effects on the host after oral treatment (Wu and Shah, 2017) preventing epilepsy, depression, diabetic complications, asthma, and cancer, as well, it plays a crucial role in the treatment of neurological disorders (Mele et al., 2019). The glutamic acid decarboxylase (GAD) enzyme, which is greatly increased under certain conditions, enables various lactic acid bacteria (LAB) to produce abundant levels of GABA, and the food industry sector has capitalized on this ability to develop functional foods rich in GABA (Diez-Gutiérrez et al., 2020). *L. brevis* strains are talented LAB that generates bioactive GABA *via* the GAD enzyme. It also plays a critical part in food processing as generally recognized as safe (GRAS) organisms as well as health-promoting probiotics and postbiotics (Wu and Shah, 2017; Oleskin and Shenderov, 2019; Abdelazez et al., 2022).

Camel milk is a functional superfood with an average of 3.1% protein, 3.5% fat, 4.4% lactose, 0.79% ash, and 11.9% total solids (Al Haj Omar and Al Kanhal Hamad, 2010). It can be used as a substitute for fruits and vegetables in arid and semi-arid areas owing to its richness in minerals (magnesium, potassium, iron, copper, zinc, and sodium) and vitamins (C, B2, A, and E) content. In addition, essential nutrients such as amino acids and fatty acids are provided to the population to enhance immunity and prevent the development of a range of disorders (Izadi et al., 2019; Seligsohn et al., 2020).

Furthermore, it contains bioactive peptides having therapeutic properties and significant benefits for human health including lactoperoxidase, hydrogen peroxide, lactoferrin, lysozyme, and immunoglobulin (El-Fakharany et al., 2012). Moreover, its anti-allergic and hypoglycemic benefits, notably among the elderly and diabetics, are attributed to its low cholesterol and sugar content (Khalesi et al., 2017). As a result, camel milk is at the forefront of more effective oral insulin administration strategies since it has higher quantities of the bioactive insulin-like protein as an anti-diabetic than other mammals milk (Agrawal et al., 2011; Khan et al., 2013; Kilari et al., 2021). It contains around 52.03 IU/ml (three times higher than cow milk) depending on the camel species, and lactation period (Abdulrahman et al., 2016; Ayoub et al., 2018).

Although there is a large body of suggestions demonstrating the ability of camel milk to balance glucose, increase insulin production, reduce insulin resistance, and improve lipid properties, as well as powerful anti-diabetes capabilities in clinical trials, *in vitro* and *in vivo* therapies (Shori, 2015). As a result, we undertook this study to investigate the current evidence on the use of camel milk for hyperglycemia and hyperlipidemia therapy, as well as GABA generation and therapeutic characteristics in regulating glucose levels in diabetic C_57_BL/6J mice for commercial use in pharmaceutical and nutritional applications.

## Materials and Methods

### Bacterial Strains and Growth Conditions

The Key Laboratory of Dairy Science provided bacterial strains *L. brevis* KLDS_1.0727_ (*LAC_S1_*) and KLDS_1.0373_ (*LAC_S2_*) strains, chemicals, and all reagents used in this study. As part of our prior investigation, 64 strains of lactic acid bacteria were screened and kept in the Key Laboratory of Dairy Science (KLDS) to determine the ideal strains that may carry the glutamic acid decarboxylase 65 (*GAD_65_*) gene and express GABA. A single colony was randomly selected and subcultured three times in De Man Rogosa Sharpe (MRS, Oxide) agar plates before being transferred to MRS broth and incubated (Sheldon Manufacturing, Inc., Shel LAB, and Cornelius, OR, United States of America) at 37°C for 18 h. Finally, cells were cultivated in MRS broth and centrifuged at 8,000 rpm for 15 min (GL-21 M High-Speed Refrigerated Centrifuge, China) to yield cell pellets that were preserved in MRS broth supplemented with 30% (v/v) glycerol and frozen at −80°C (MDF4V; Panasonic, Tokyo, Japan).

### PCR Amplification Analyses of *GAD_65_*

*Lactobacillus brevis* KLDS_1.0727_ (*LAC_S1_*) and KLDS_1.0373_ (*LAC_S2_*) strains were inoculated (1% v/v) in MRS broth enhanced with 1% L-monosodium glutamate (Sigma, United States of America), and cultured anaerobically at 37°C overnight using a glove chamber (Sheldon, United States of America) provided incubation atmosphere with a gas mixture consisting of 90% nitrogen, 5% hydrogen, and 5% carbon dioxide to help strains to generate postbiotic GABA.

The TIANamp Bacteria DNA Kit (Tiangen Biotech Ltd., Beijing, China) was used to get the Genomic DNA under the manufacturer’s extraction guidelines. Furthermore, DNA was extracted once the bacterial cell wall was ruptured with lysozyme. PCR amplification of the *gad* fragments was amplified by PCR (GeneAmp PCR System 9,700 thermal cycler Applied Biosystem, United States of America) using primers constructed with the oligo 6 software (Molecular Biology Insights, Inc. DBA Oligo, Inc.) as follows:

*gadF*:5′CCTCGAGAAGCCGATCGCTTAGTTCG-3′; *gadR*:5′TCATATTGACCGGTATAAGTGATGCCC-3.

The PCR technique stages have been previously described (Abdelazez et al., 2018). 50 μl of PCR mixture were divided as (1.0 μl of DNA template, two primer pairs (ComateBio Custom Primers, Jilin, Changchun, China), *gadF*/*gadR* (10 μmol/l); 2.0 μl, DNA Polymerase (2.5 U/μl); 0.5 μl, 10 × Taq Buffer DNA polymerase (Sigma, United States of America); 5.0 μl, 2′-deoxynucleoside 5′ triphosphate (dNTPs), (2.5 mM) 4.0 μl, and ddH_2_O 35.5 μl). The PCR techniques were carried out at the denaturation stage (95°C for 5 min). Then came the annealing stage was performed (95°C for 30 s, 55°C for 1.30 min). Finally, the elongation stage was performed (1.30 min at 72°C). A further extension session of 10 min at 72°C was added after 30 cycles.

The amplification products were separated by electrophoresis on a 1.5% agarose gel (Sigma, United States of America) in TAE buffer (0.04 M Tris-acetate, 1 mM EDTA, pH 8). For 1 h, the gel was run at a constant voltage of 100 V then the gels were stained with 0.2 μg/ml ethidium bromide for 15 min. A UV light transilluminator was used to visualize the PCR products. To determine band sizes, a 100-base pair DNA ladder (Gibco-BRL, Grand Island, New York, NY, United States of America) was loaded into the first lane of each gel. A gel documentation system was used to photograph the gels under UV light (Bio-Rad, Hercules, CA, United States of America). KLDS_1.0727_ and KLDS_1.0373_ were compared using sequences taken from the NCBI genome database.^1^

### Potential Probiotic Functions of KLDS_1.0727_ and KLDS_1.0373_ Strains

Before using *L. brevis* strains *in vivo*, several *in vitro* experiments were previously performed to examine their potential ability to survive in harsh GIT conditions such as (pH, bile tolerance, antagonism against pathogens, resistance to several types of antibiotics, viability in simulating gastrointestinal juice, etc.) as free pellets or freeze-dried strains were performed previously by Abdelazez et al. (2018, 2022).

### *In vivo* Experiments

#### The Experiment Protocol

The *in vivo* experiment was carried out in accordance with the Northeast Agricultural University’s institutional animal care and use committee guidelines, as well as the China Ministry of Science and Technology Guide for the Care and Use of Laboratory Animals, under the authorized protocol number specialized pathogen-free rodent management (SRM)-06.

#### Preparation of Oral Gavage Inoculums

For 4 weeks, *LAC_S1_* and *LAC_S2_* were subcultured in MRS broth (1% v/v) at 37°C. Strain pellets were harvested after 18 h, particularly in the third generation, by centrifuging at 2,500 rpm for 10 min at 4°C. The bacterial pellets were resuspended in sterile PBS after three rinses in phosphate buffer saline (PBS) at a concentration of 5 × 10^8^ CFU/ml, and the vials inoculum comprised 250 μl/mouse. Raw camel milk samples were collected from a bulk tank containing milk from several animals in Taif, Saudi Arabia. The samples were delivered to the animal house lab and stored frozen in a sterile icebox until the study began. One vial of camel milk inoculum containing 100 μl/mouse was used in each of the control and diabetic mice therapies.

#### The Experimental Design

The experiments were designed on seven groups (*n* = 8 mice/group; C_57_BL/6 J, 16–25 g/mice, Vital River Laboratory Animal Technology Company, Beijing, China, approval number SCXK, JING, 2012-0001). Mice were adapted for 1 week in a well-ventilated cage with access to potable water and standard pathogen-free rodent chow in a regular animal house (23 ± 2°C, relative humidity 50 ± 20%, 12 h light/dark). Except for the control group (*Cont*), which was given 250 μl sterile PBS/day, and the camel milk control (*Ca_Cont_*), which was fed 100 μl of raw camel milk. The other five treated groups were injected with streptozotocin (*STZ*, 180 mg/kg; Sigma, United States of America) that was newly formulated in 50 mM sodium citrate buffer (pH 4.5) and delivered subcutaneously within 10–15 min after disintegrating for 1 day (Wu et al., 2011).

Three days later after receiving streptozotocin, animals having glucose concentrations of more than 7 mmol/l were deemed diabetic, while mice with low glucose levels were eliminated. There were five groups of diabetic mice in the study. Streptozotocin control (*STZ*) group that injected intraperitoneally with 180 μl streptozotocin only. (*INS_STZ_*) was daily given insulin subcutaneously injection (Sigma, United States of America) and fed rodent chow for 4 weeks at a dosage of (0.5 unit/kg body weight). Two groups of *L. brevis* strains (*LAC_S1_* and *LAC_S2_*) were dissolved in buffered saline and orally gavage at a dose intake of 250 μl × 10^5^ CFU/ml/day, and one group was gavage orally 100 μl/day of camel milk (*Ca_STZ_*), using a stainless oral needle.

#### Evaluation of Body Weight and Hyperglycemia in *STZ*-Induced Diabetic Mice Weekly

Weekly body weight was conducted using an electronic balance (Hogentogler & Co. Inc. United States of America). Furthermore, fasting blood glucose after 12 h and postprandial blood glucose after 2 h were determined using a glucometer (Contour H Meter, Bayer HealthCare LLC, United States of America) in blood samples taken from an ophthalmic vein. The postprandial average of glucose levels was calculated using the equation suggested by Abdelazez et al. (2018).


Average of glucose levels=Postprandial2h−Fasting12h

#### Evaluation of Hyperlipidemia and Glucose Levels in Blood Plasma

All blood plasma parameters were performed after being humanely sacrificed under diethyl anesthesia and all parameters were assessed in triplicate using the Beckman Coulter UniCel D × C 800 analyzer (Beckman Coulter, Miami, FL, United States of America) which is available at the Center of Drug Safety Evaluation, Heilongjiang University of Chinese Medicine, Harbin, China. Overnight fasting blood samples were collected from all groups and allowed to coagulate at 4°C before being centrifuged at 12,000 rpm/10 min. Triglyceride (TG), total cholesterol (CHOL), high-density lipoprotein cholesterol (HDL), and low-density lipoprotein cholesterol (LDL) levels were measured to detect hyperlipidemia. Also, glucose (GLU) and Mg^+2^ were evaluated.

#### Evaluation of Liver and Renal Functions

To investigate liver functions, serum alanine aminotransferase (ALT), aspartate transaminase (AST), total bile acid (TBA), albumin (ALB), globulin (GLUB), and total protein (TP) levels were assessed. As well, uric nitrogen (BUN), creatinine (CREA), and uric acid (URIC) levels were measured to assess renal function.

#### Assessment of Blood Serum Insulin

ELISA kits (Meimian Biotech Co., Ltd., Yancheng, China) were used to detect serum insulin following the insulin kit manufacturer’s guidelines.

#### Histopathological Analysis

The liver, pancreas, kidney, and spleen of euthanized mice had been aseptically removed from all test groups (Chen et al., 2012). After being placed in a 10% formalin solution, the organs were washed in PBS, followed by rinses with graduated alcohol concentrations of (75–100%), as well as xylene 100%. The specimen was then paraffin-fixed and sliced into 5–10 μm thickness before being stained with hematoxylin and eosin (Solarbio, China). The slices were inspected at 100 magnifications using light microscopy (Olympus, Japan). Three photos were taken of different organ sections then the average of the affected area/mouse was determined.

### Statistical Analysis

All experiments were performed in triplicate using independent tests. The obtained data were analyzed using one-way analysis of variance (ANOVA) using GraphPad Prism 8.1 (GraphPad Software Inc, San Diego, CA, USA) to create graphs and calculate statistics, and the results were represented as Mean ± SD. Values of *p* < 0.05 were considered to be statistically significant.

## Results

### Evaluation of *GAD_65_* 16S rRNA Sequences

The quantity of GABA generated by selected strains was prior determined using PCR and HPLC. The results of the HPLC quantitative analysis revealed that two (KLDS_1.0727_ and KLDS_1.0373_) *LAC_S1_* and *LAC_S2_* had the best GABA-producing ability with concentrations of 1.98 ± 0.07 and 0.05 ± 0.05 g/l, respectively, and a long band of 1.407 bp (Abdelazez et al., 2018, 2022).

### Glucose Determination During 4 Weeks

**Figure 1:**
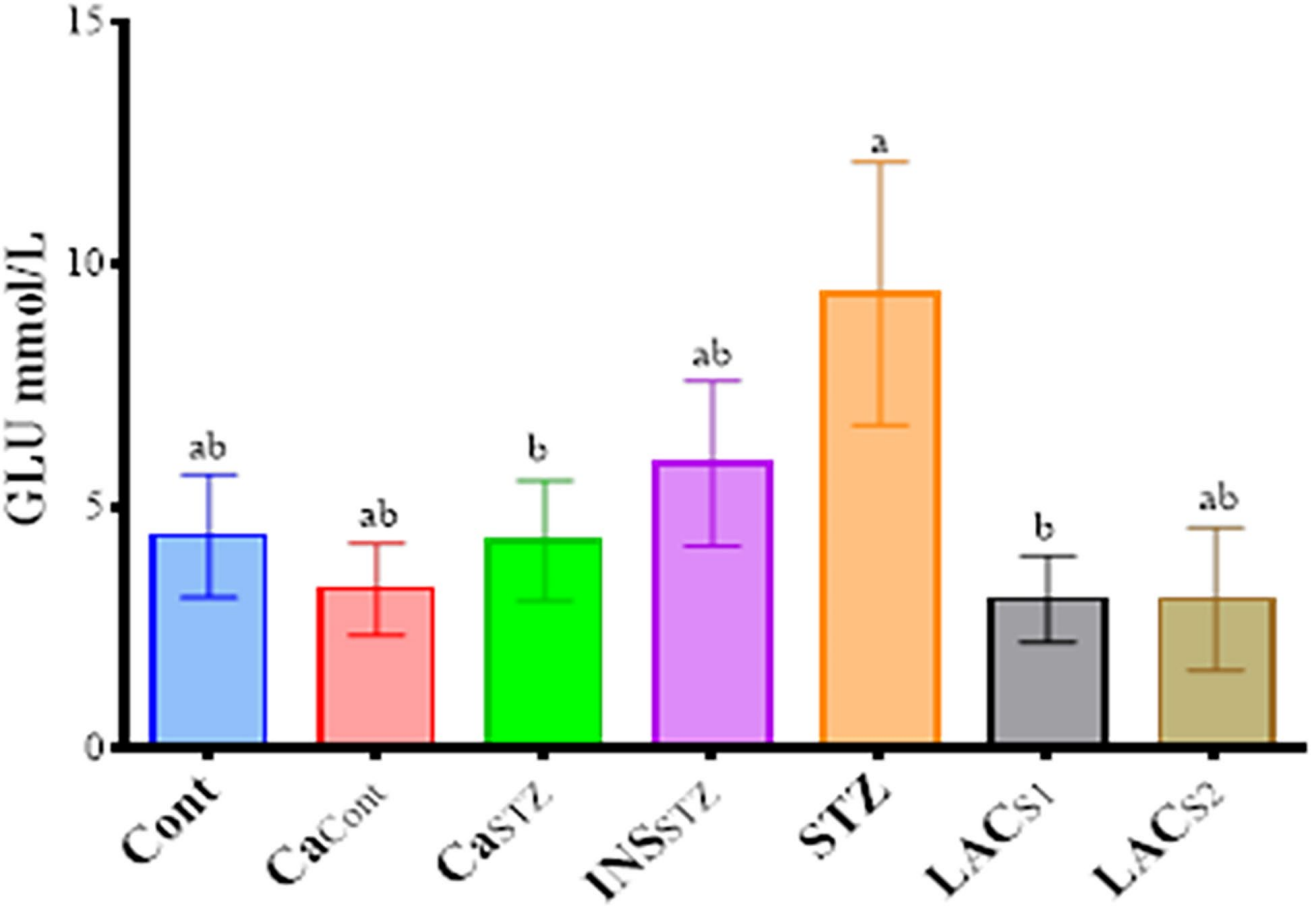
Glucose levels (mmol/l) during 4 weeks. *Cont*, control; *Ca_Cont_*, camel milk control; *Ca_STZ_*, camel milk with streptozotocin; *INS_STZ_*, insulin with streptozotocin; *STZ*, streptozotocin; *LAC_SI_*, KLDS_1.0727_ with streptozotocin; *LAC_S2_*, KLDS_1.0373_ with streptozotocin. Result are given in triplicate as mean ± SD. Different letters indicate significant differences among groups (*p* < 0.05).

The results demonstrated the significant differences (*p* < 0.05) between treatments during the first week, the *INS*_STZ_ group showed severe glucose concentration reduction, and this trend continued to drop during the third and fourth weeks. Figure 1 shows the glucose values of *INS*_STZ_ (− 5.12 ± 2.12, and − 7.67 ± 2.40 mmol/l) respectively and it exhibited the lowest postprandial glucose concentrations when compared to *STZ* (6.75 ± 3.75 and 6.77 ± 0.97 mmol/l) in the first and the 4th weeks, respectively. However, *Ca_Cont_* and *Ca*_STZ_ had (1.97 ± 1.31 and 1.87 ± 0.59 mmol/l) in the first week and (5.17 ± 0.18 and 5.82 ± 1.07 mmol/l) respectively in the fourth week exhibited the lowest GLU concentrations in the first week of feeding camel milk when compared to *Cont* (3.55 ± 1.44 mmol/l). While, *LAC_S1_* and *LAC_S2_* showed (4.25 ± 0.83 and 5.66 ± 2.84 mmol/l) respectively. All treatments had higher postprandial glucose concentrations than *Cont* (4.15 ± 0.14 mmol/l) after 4 weeks of therapy.

### Average of Bodyweight During 4 Weeks

**Figure 2:**
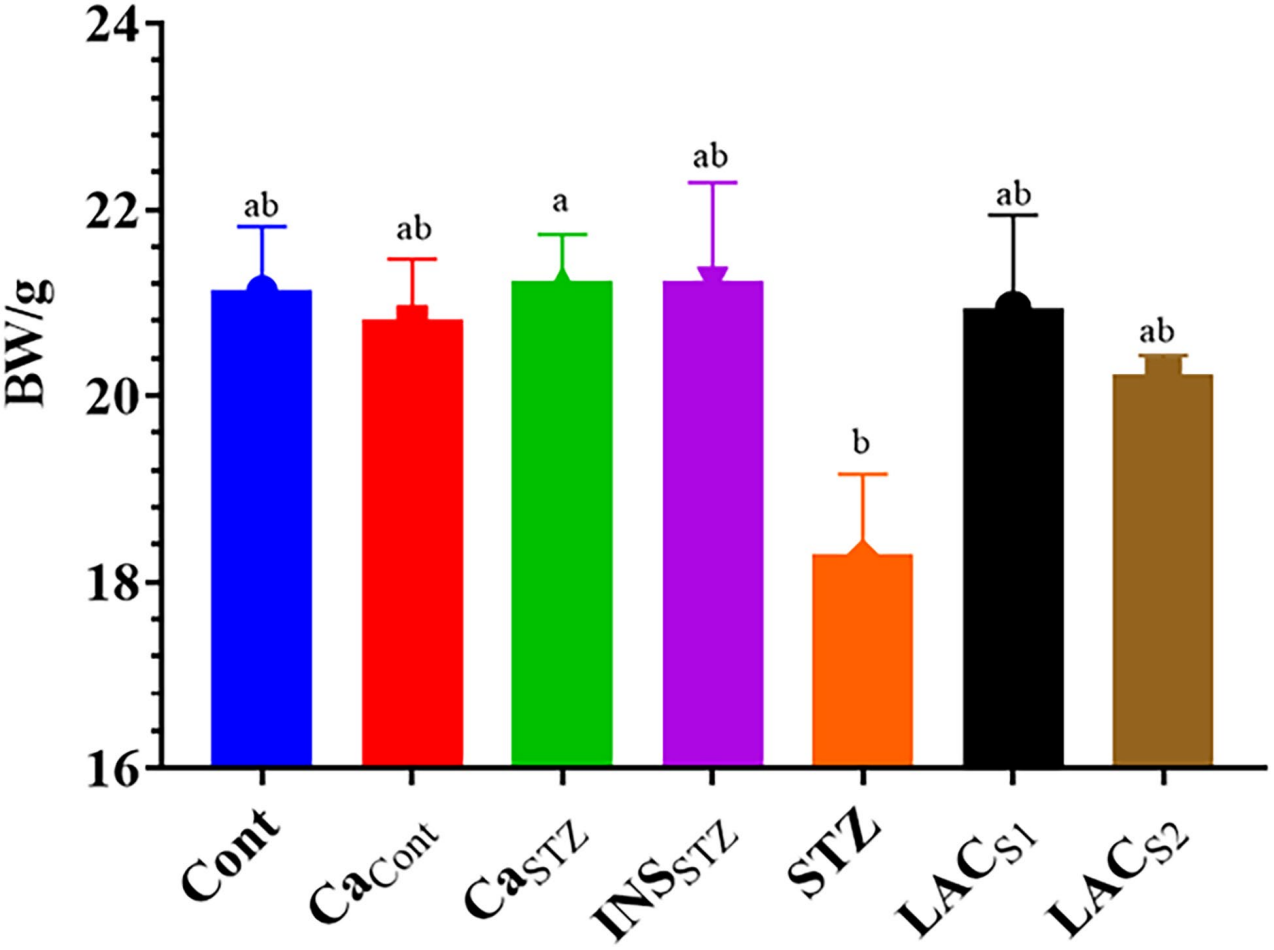
Average of bodyweight/g during 4 weeks. *Cont*, control; *Ca*_*Cont*_, camel milk control; *Ca_STZ_*, camel milk with streptozotocin; *INS_STZ_*, insulin with streptozotocin; *STZ*, streptozotocin; *LAC_SI_*, KLDS_1.0727_ with streptozotocin; *LAC_S2_*, KLDS_1.0373_ with streptozotocin. Result are given in triplicate as mean ± SD. Different letters indicate significant differences among groups (*p* < 0.05).

Significant differences (*p* < 0.05) were seen across groups during 4 weeks of treatment. Figure 2 shows that *Cont*, *Ca_STZ_*, and *INS_STZ_* had the highest average body weights (21.13 ± 0.68, 21.22 ± 0.50, and 21.21 ± 1.07 g) respectively. While *STZ* showed the lowest average body weight 18.30 ± 0.85 g.

### Hyperlipidemia Parameters, Glucose, and Mg^+2^ Levels Analysis in Blood Plasma

**Figure 3:**
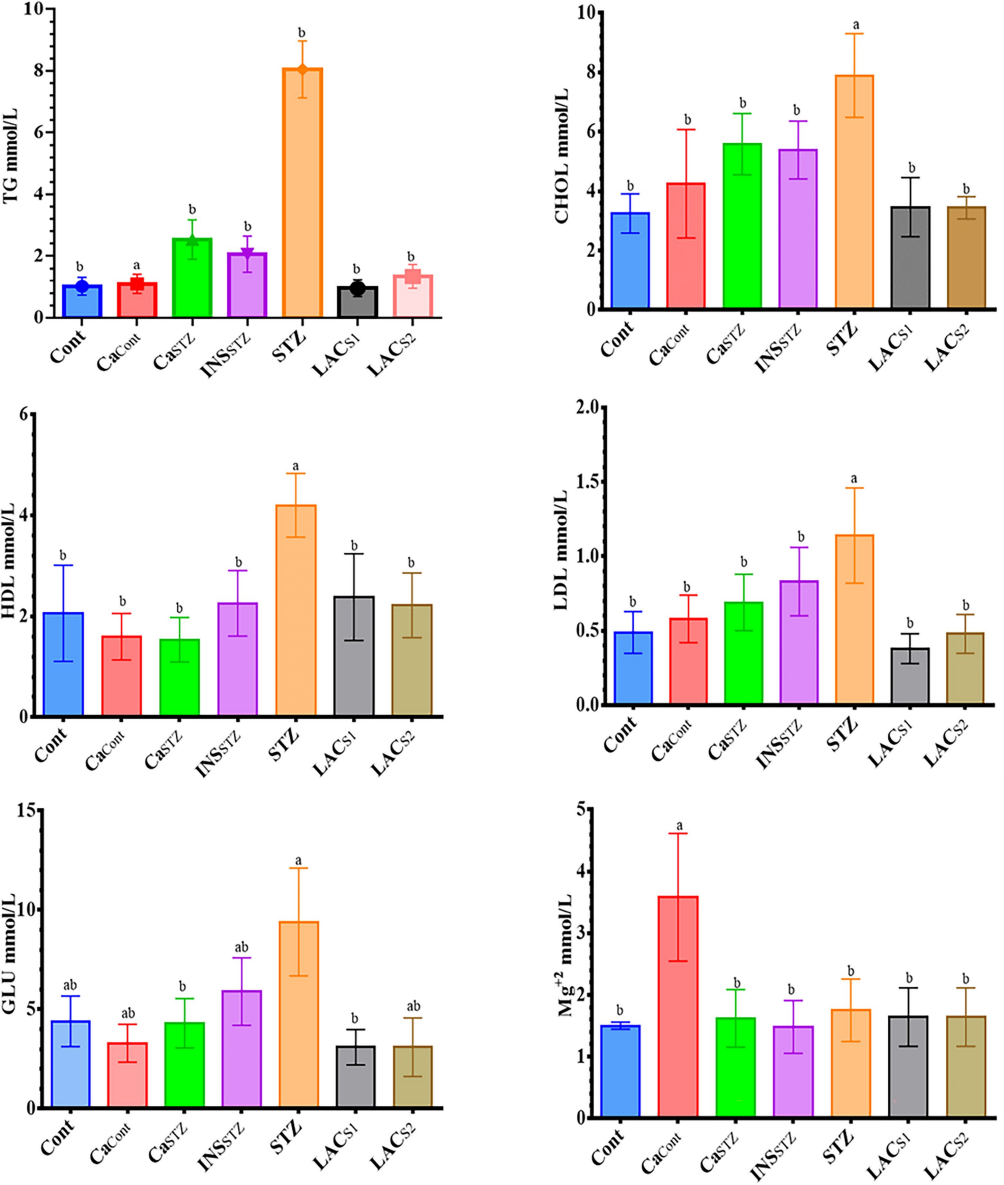
Hyperlipidemia parameters, glucose and Mg^+2^ level analysis in blood plasma. *Cont*, control; *Ca_Cont_*, camel milk control; *Ca_STZ_*, camel milk with streptozotocin; *INS_STZ_*, insulin with streptozotocin; *STZ*, streptozotocin; *LAC_SI_*, KLDS_1.0727_ with streptozotocin; *LAC_S2_*, KLDS_1.0373_ with streptozotocin. Result are given in triplicate as mean ± SD. Different letters indicate significant differences among groups (*p* < 0.05). TG, triglycerides; CHOL, total cholesterol; HDL, high-density lipoprotein cholesterol; LDL, low-density lipoprotein cholesterol; GLU, glucose.

Figure 3 displays hyperlipidemia, glucose, and Mg^+2^ in blood plasma, which includes TG, CHOL, HDL, and LDL. In all treatment groups, the results revealed a high significance (*p* < 0.05). The greatest TG content was found in *STZ* (8.05 ± 0.92 mmol/l), whereas *LAC_S1_* had the lowest TG level (0.96 ± 0.27 mmol/l). In addition, the greatest concentration of CHOL was found in *STZ* (7.89 ± 1.41 mmol/l), while, *Cont* showed the lowest CHOL concentration (3.25 ± 0.66 mmol/l). Furthermore, *STZ* had the highest HDL and LDL values (4.2 ± 0.63, and 1.14 ± 0.32 mmol/l) respectively, whereas *Ca_STZ_* and *Ca*_*Cont*_ had the lowest HDL (1.54 ± 0.44 and 1.6 ± 0.5 mmol/l) respectively. Furthermore, *LAC_S1_* had the lowest LDL concentration (0.38 ± 0.10 mmol/l). Also, there was a significant difference in GLU levels (p < 0.05), *STZ* had the greatest (9.4 ± 2.71 mmol/l) and *LAC_S1_* and *LAC_S2_* showed the lowest GLU levels (3.1 ± 0.89 and 3.1 ± 1.47 mmol/l), while *Ca*_*Cont*_ showed the highest Mg^+2^ level (3.58 ± 1.03 mmol/l) and *INS_STZ_* showed the lowest level (1.48 ± 0.42 mmol/l).

### Determination of Insulin Blood Plasma

**Figure 4:**
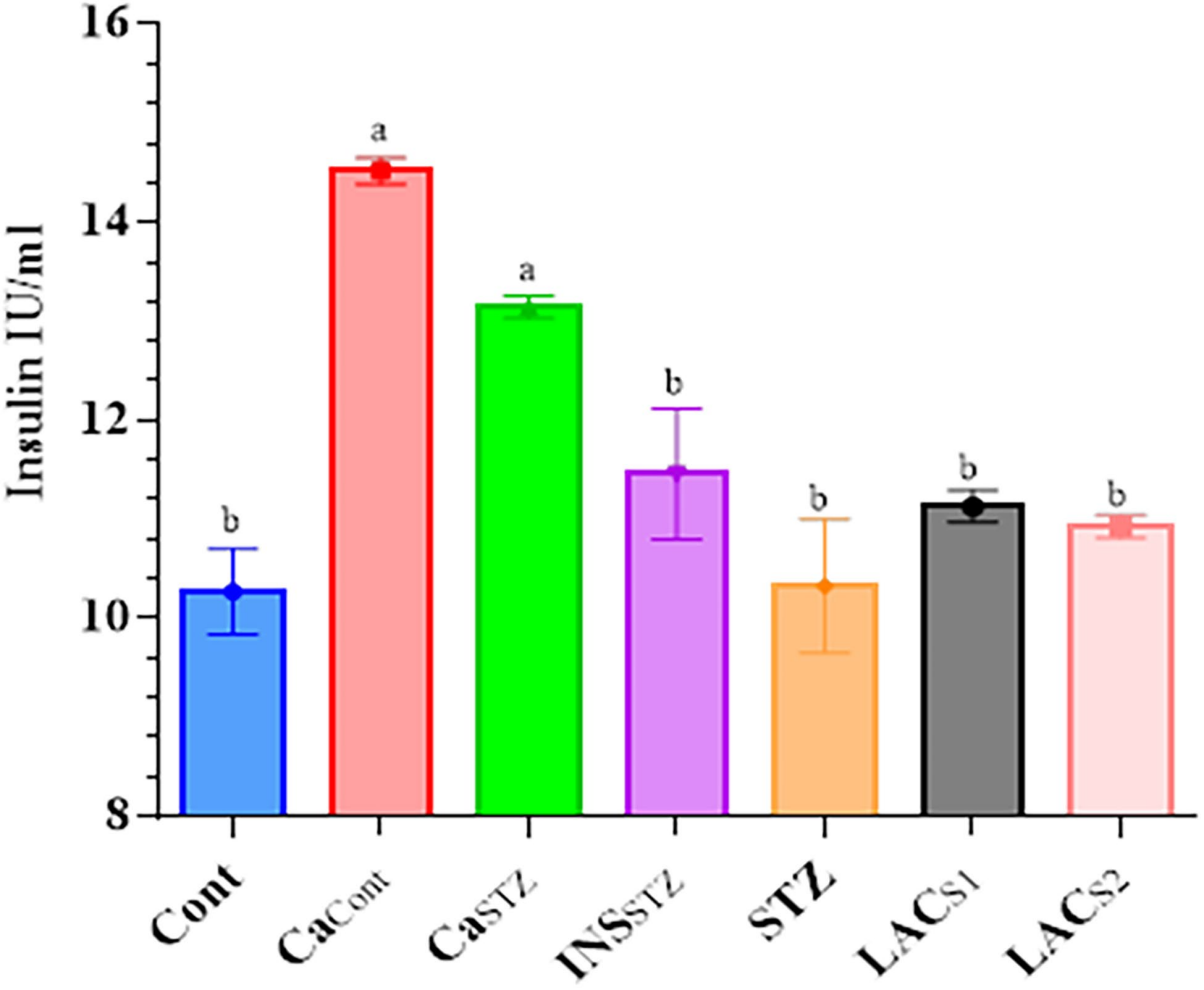
Determination of insulin blood plasma. *Cont*, control; *Ca_Cont_*, camel milk control; *Ca_STZ_*, camel milk with streptozotocin; *INS_STZ_*, insulin with streptozotocin; *STZ*, streptozotocin; *LAC_SI_*, KLDS_1.0727_ with streptozotocin; *LAC_S2_*, KLDS_1.0373_ with streptozotocin. Result are given in triplicate as mean ± SD. Different letters indicate significant differences among groups (*p* < 0.05).

Figure 4 depicts the insulin blood plasma concentrations, which showed no significant difference (*p* < 0.05). *Ca*_*Cont*_ and *Ca_STZ_* had the highest insulin concentrations (14.14 ± 0.11 and 13.52 ± 0.13 IU/ml) respectively, while *Cont* had the lowest level (10.27 ± 0.43 IU/ml).

### Assessment of Liver Functions

**Figure 5:**
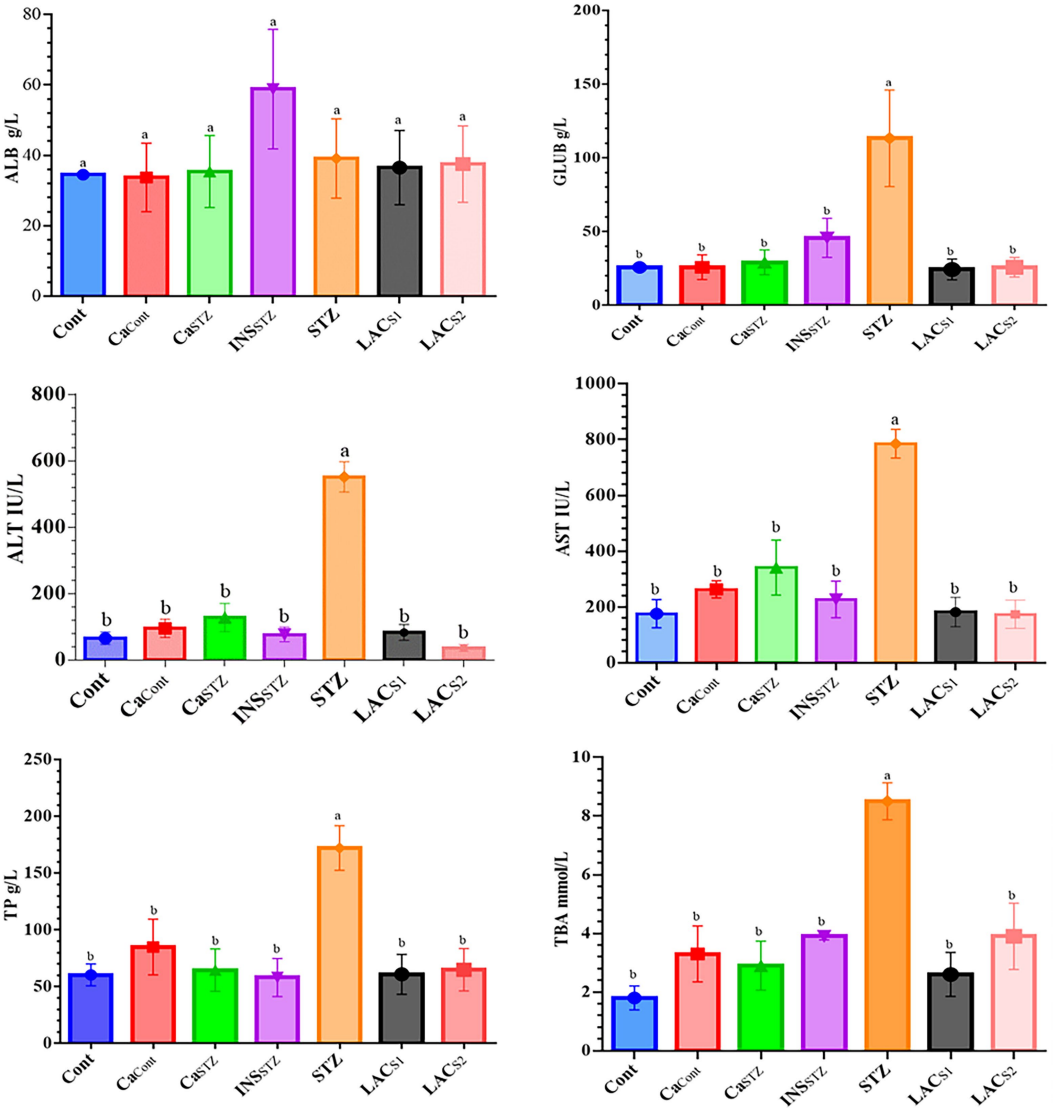
Assessment of liver functions. *Cont*, control; *Ca_Cont_*, camel milk control; *Ca_STZ_*, camel milk with streptozotocin; *INS_STZ_*, insulin with streptozotocin; *STZ*, streptozotocin; *LAC_SI_*, KLDS_1.0727_ with streptozotocin; *LAC_S2_*, KLDS_1.0373_ with streptozotocin. Result are given in triplicate as mean ± SD. Different letters indicate significant differences among groups (*p* < 0.05). ALB, albumin; GLUB, globulin; ALT, serum alanine aminotransferase; AST, aspartate transaminase; TP, total protein; TBA, total bile acid.

Figure 5 indicates that *INS_STZ_* resembled *Cont*, which had the lowest TP concentrations (58.1 ± 16.77 and 60.3 ± 9.63 g/l) respectively compared to *STZ*, which had the greatest TP level (172.1 ± 19.68 g/l). Also, it was significant differences at (*p* < 0.05). Conversely, *Ca*_*Cont*_ had the lowest ALB concentration (33.7 ± 9.72 g/l), whereas *INS_STZ_* had the highest ALB level (58.8 ± 16.97 g/l). The highest GLUB concentration was found in *STZ* (113.3 ± 32.70 g/l), whereas the lowest was found in *LAC_S1_* (24.47.04 g/l). *Cont* showed the Lowest TBA value (1.8 ± 0.41 mmol/l), while the greatest concentration was observed in *STZ* (8.5 ± 0.63 mmol/l). Furthermore, *STZ* showed the highest levels of ALT (551 ± 45.66 IU/l). While *LAC_S1_* had the lowest level (36.0 ± 10.39 IU/l). Also, *STZ* displayed the highest concentration of AST (785 ± 51.28 IU/l) while *Cont* and *LAC_S2_* showed the lowest concentration (176 ± 50.80 and 174 ± 50.56 IU/l) respectively.

### Assessment of Renal Functions

**Figure 6:**
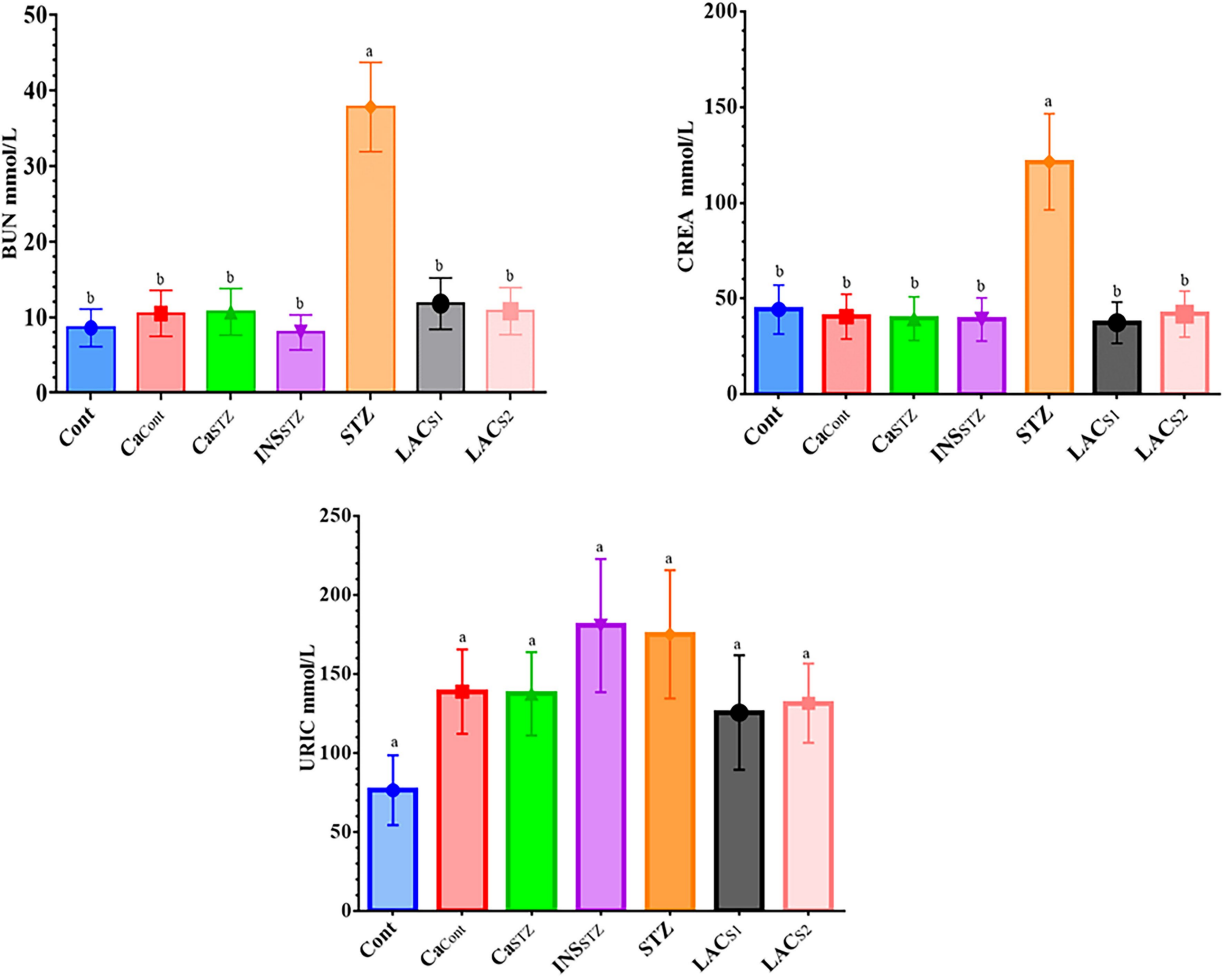
Assessment of renal functions. *Cont*, control; *Ca_Cont_*, camel milk control; *Ca_STZ_*, camel milk with streptozotocin; *INS_STZ_*, insulin with streptozotocin; *STZ*, streptozotocin; *LAC_SI_*, KLDS_1.0727_ with streptozotocin; *LAC_S2_*, KLDS_1.0373_ with streptozotocin. Result are given in triplicate as mean ± SD. Different letters indicate significant differences among groups (*p* < 0.05). BUN, uric nitrogen; CREA, creatinine; URIC, uric acid.

The findings in Figure 6 showed that *STZ* had the greatest concentrations of BUN, CREA, and URIC (37.8 ± 10.91, 121.5 ± 35.07, and 180.6 ± 52.13 mmol/l) respectively, whereas *Cont* had the lowest concentrations of BUN, CREA, and URIC (8.6 ± 2.48, 39.5 ± 11.40 and 76.5 ± 22.08 mmol/l) respectively. There was no abnormal elevation in URIC at (*p* < 0.05). However, BUN and CREA indicated a significant difference at (*p* < 0.05).

**Figure 7:**
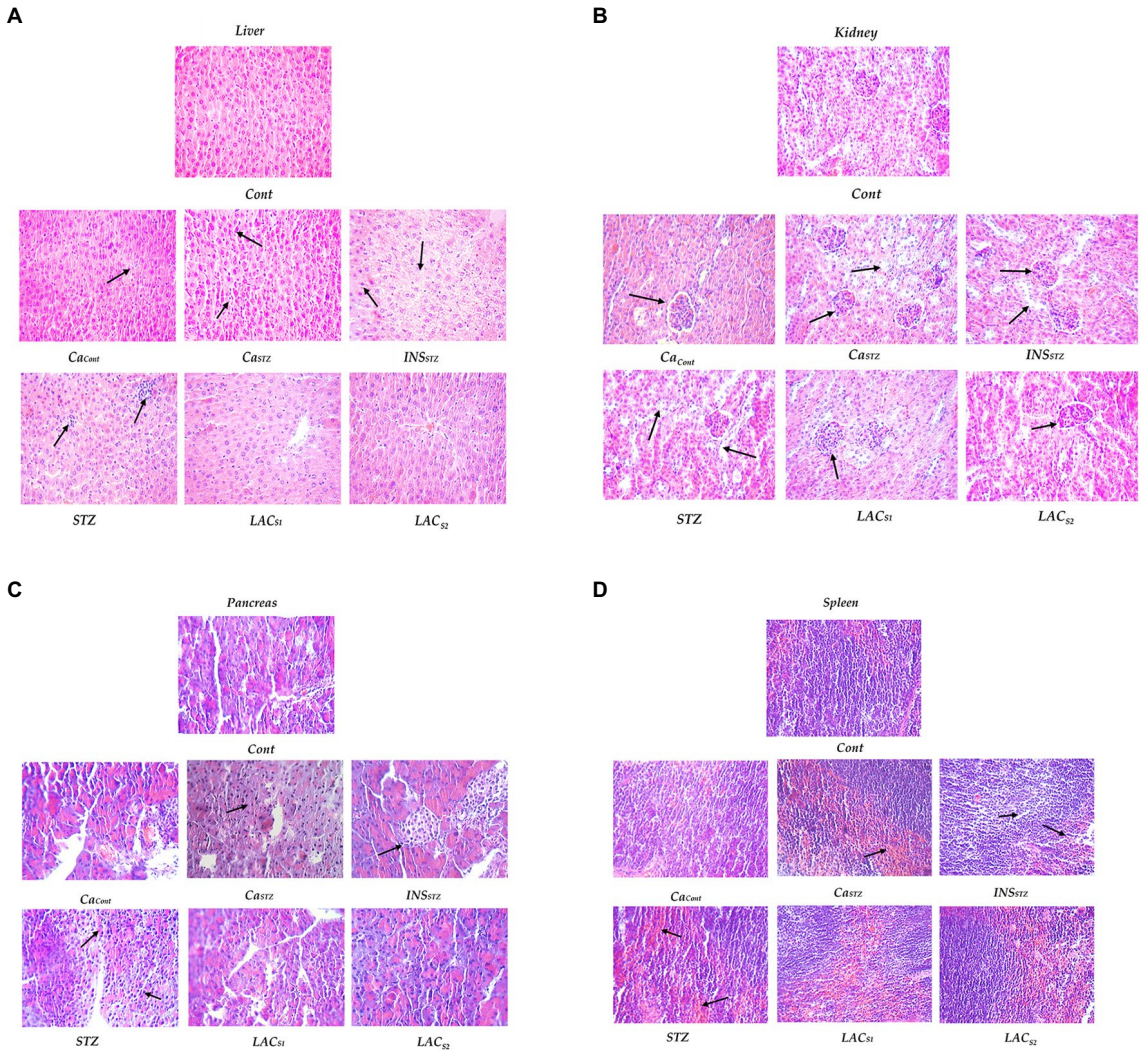
Histopathological evaluation. *Cont*, control; *Ca_Cont_*, camel milk control; *Ca_STZ_*, camel milk with streptozotocin; *INS_STZ_*, insulin with streptozotocin; *STZ*, streptozotocin; *LAC_SI_*, KLDS_1.0727_ with streptozotocin; *LAC_S2_*, KLDS_1.0373_ with streptozotocin. **(A)** Lever; **(B)**) Kidney; **(C)** Pancreas and **(D)** Spleen.

Briefly, using *Lactobacillus brevis* strains and camel milk had a clear influence on fasting and postprandial glucose levels, as well as body weight, in diabetic mice for 4 weeks. Furthermore, the impact of GABA and camel milk was more visible in blood plasma by comparing insulin concentration, liver and kidney function, as well as cholesterol compounds and glucose levels in streptozotocin-treated mice to control groups.

### Histological Evaluation

Figures 7A–D depicts a histological examination of (the liver; kidney; spleen, and pancreas). *Cont* shows that the liver had no aberrant shape, cytoplasmic nuclei was clean borders, and there was no congestion, denatured fat, or inflammatory cell infiltration. Also, the renal glomeruli were normal, with no shrinkage or atrophy, and the renal tubules were also normal. The spleen was a normal proportion of red pulp and white marrow cells, normal splenic trabecula structure, and lymphocytes with no hyperemia or fibrosis. Pancreatic cells and vessels, which were typical islet cells, did not shrink and showed no signs of fibrosis or expansion.

*Ca*_*Cont*_ reveals no apparent abnormalities or sporadic liver nuclei pyknosis and no inflammatory cell infiltration. Mild fatty deterioration was seen. Meanwhile, there was some glomerular atrophy and renal tubular fusion in the kidney. The spleen had enhanced phagocytic activity as well as lymphopenia. On the other hand, the pancreas shows no abnormalities.

*Ca_STZ_* indicates no apparent abnormalities or sporadic hepatic nuclei pyknosis and no inflammatory cell infiltration in the liver. Meanwhile, the kidney was atrophied and glomerular shrinkage was visible in the renal tube. The spleen displayed enhanced phagocytic and lymphopenia, whereas the pancreas was normal.

Streptozotocin (*STZ*) showed hepatic steatosis, and inflammatory cells collected and spread in heaps. While renal glomeruli shrunk or atrophied, renal tubules were deemed normal, and no inflammatory cell infiltration or fibrosis was observed. Meanwhile, the spleen indicated that the number of lymphocytes had reduced and their structure had been disrupted. Pancreatic cells show vacuolar degeneration and islet atrophy.

Insulin with Streptozotocin (*INS*_*STZ*_) demonstrated that the liver had moderate fatty degeneration and was slightly denatured. Renal glomeruli shrunk or atrophied, and epithelial cells fell off renal tubules. The spleen showed increased trabecular and lymphatic decrease, as well as structural loss. Pancreatic islet cells shrank significantly, and there was extensive vacuolar degeneration.

*LAC_S1_* and *LAC_S2_* indicate that the liver has normal morphology. There were fewer lymphocytes in the spleen tissue. There had been no abnormalities in pancreatic cells it had not been altered. The glomerulus was similarly normal, and the renal tubes were slightly fused but generally normal.

## Discussion

A dysbiosis of the gut microbiota can lead to a variety of disorders, including type 1 diabetes and cancer (Carding et al., 2015). Diabetes type 1 can be diagnosed by taking measurements of both body weight and blood glucose levels. As a result, it is now recognized as a serious metabolic disorder defined by chronic hyperglycemia caused by insufficient insulin secretion (Di Martino et al., 2021).

Postbiotics are substrates that are created or synthesized by microbial metabolic activities and have a direct or indirect beneficial impact on the host (Żółkiewicz et al., 2020; Salminen et al., 2021). The capability of LAB to synthesize GABA differs between species and even within species, and it may be related to glucose metabolism and growth rate (Cataldo et al., 2020). For several lactic acid bacteria such as *Lactobacillus rhamnosus*, *Lactobacillus reuteri*, and *Bifidobacterium infantis* postbiotic supplementation increased GABA activity and improved oxidative balance (Bagheri et al., 2019). Also, *Lactobacillus brevis* can generate GABA and control the increase of glucose levels in diabetic mice (Abdelazez et al., 2022). Furthermore (Tian et al., 2004), showed that administering the GABA molecule as a medicinal agent can reduce inflammatory responses and the development of pre-diabetes. Wan et al. (2015) discovered that the GABA molecule has a regulatory effect on human diabetic islands, namely inhibiting insulitis and systemic inflammatory cytokine production.

A blood test reference range is a set of numbers that a health practitioner uses to evaluate a range of medical test results from clinical specimens. Several factors, including age, gender, fitness, and ethnicity, as well as analytical procedures and measuring units, all influence the reference range of the results achieved. Individual results should always be interpreted in light of the test laboratory’s testing facility (Virani et al., 2021). Notably, we used these standard references to evaluate the results and compared the other groups with the control group.

Glucose levels are lower before the first meal and rise for 1–2 h after eating. Extra blood glucose levels may suggest the existence of chronic diseases. The findings are similar to those of (Rees et al., 2016), who stated that diabetes was considered as fasting blood glucose levels of greater than 7 mmol/dl to 11.1 mmol/dl or higher with hyperglycemia symptoms. Sacks et al. (2011), reported the GLU varied between 3.57 and 6.12 mmol/lb. As a result, *INS_STZ_* had the lowest glucose level due to the quick insulin injection, while *STZ* had the highest glucose concentration. Whereas the remainder of the test groups remained within the standard range of glucose over the 4 weeks of treatment.

The results are consistent with the findings of (Graham et al., 2007) who revealed that insulin is the most important factor in improving plasma glucose homeostasis and it ranges between 3 and 19 IU/ml in blood plasma.

Changes in ALT and AST levels are likely the most commonly employed in clinical diagnosis and research concerning liver damage and tissue activities. According to Contreras-Zentella and Hernández-Muñoz (2016), a rise or reduction in AST and ALT levels might occur as a result of liver injury, which causes changes in cell membrane permeability. The streptozotocin injunction may explain the dramatic increase in AST and ALT levels in the *STZ* group therefore the results were consistent with Saravanakumar et al. (2021), who found that (ALT and AST), and TBA levels were (1.0–40.0 IU/lb) and 0.01–20.0 mmol/lb, respectively, and who discovered that streptozotocin raises AST, ALT, and ALP levels in blood plasma. Also, the findings were in agreement with those of Newsome et al. (2018) who found that the normal ranges for TP, ALB, and GLUB were 60.0–80.0 g/dl, 35.0–55.0 g/dl, and 25.0–40.0 g/dl, respectively.

Renal dysfunction is caused by hyperuricemia, hyperglycemia, and hypertension. Also, diabetes kidney failure is one of the leading causes of mortality. Therefore, kidney function tests are the most important indicators of renal activity, vitality, and general health (Stevens and Levin, 2013).

Ibraheem et al. (2016) displayed that the reference range for BUN was 1.07–7.14 mmol/lb, CREA was 53.0–132.0 mmol/lb, and the URIC was 142.0–401.0 mmol/l. The results demonstrate that CREA levels in the therapy groups were within the safe range of CREA, which is one of the most significant kidney function tests whose results are used to evaluate renal functioning. Furthermore, serum creatinine measurement is a straightforward test that may be performed to diagnose acute renal damage or dehydration, and it is the most frequent indication of kidney function (Harloff et al., 2021).

Camel milk has an insulin-like protein that mimics the interaction of insulin with its receptors, is resistant to proteolysis, and has higher storage buffering capacity than other ruminants milk (El-Sayed et al., 2011; Swelum et al., 2021). It is also encapsulated in nanoparticles such as lipid vesicles and rapidly absorbed into the circulation. As a consequence, innovative oral insulin treatment strategies might be investigated (Ashraf et al., 2021). Camel milk has a high concentration of zinc, which is essential for the activity of insulin production in pancreatic β-cells (Deeba et al., 2020). Camel milk’s functional purpose is not limited to stimulating, increasing, and boosting insulin production; it also aids in the development and improvement of pancreatic β-cells efficiency (Ayoub et al., 2018).

The findings were compared to those of investigations that evaluated the effect of camel milk intake on type 1 diabetes mellitus (Dikhanbayeva et al., 2021; Manaer et al., 2021), regardless of whether the clinical treatment period was 3 or 6 months. Patients who consumed camel milk for a short or long period exhibited significant decreases in blood glucose levels as well as insulin requirements. Furthermore, they evaluated insulin sensitivity and glycemic control in individuals with type 1 and 2 diabetes, revealing that fasting and postprandial blood glucose levels and insulin needs decreased considerably (Mudgil et al., 2018).

Camel milk supports decreased LDL cholesterol levels, hypoglycemia, a boost in immunostimulants, anticancer, and antibacterial agents, and improved consumer overall health (Esraa et al., 2016).

Importantly, the obtained results were consistent with earlier investigations in diabetic rabbits, where regular camel milk intake reduced oxidative damage (Aqib et al., 2019), whereas, raw camel milk reduces blood glucose levels in diabetic rats by 55% compared to 43% for raw cow milk (El-Zahar et al., 2021). Khan et al. (2013) reported an almost 30% decrease in blood glucose levels in *STZ*-induced rats given camel milk for 6 weeks, from 560 to 235 mg/dl compared to rats given buffalo milk or cow’s milk.

Camel milk feeding lowers the percentage of lipid peroxide (malondialdehyde levels) and catalase activity while raising glutathione and superoxide dismutase (SOD) levels in *STZ*-induced diabetic rabbits. Furthermore, camel milk has a significant influence on insulin receptor function and glucose transfer in insulin-sensitive organs such as the liver and pancreas, both of which play key roles in the maintenance of blood glucose homeostasis (Al-Hashem et al., 2009). On the contrary, Fallah et al. (2020) discovered that the hypoglycemic benefits of camel milk on type 2 diabetes patients can only be sustained when combined with diabetic medicines. Furthermore, Ejtahed et al. (2015) discovered no change in glucose, lipids, or blood pressure parameters after consuming camel or cow’s milk. Therefore, we conducted these experiments to demonstrate the effect of regularly consuming camel milk as a type of traditional functional food, as well as expanding its use with fermented foods that contain *L. brevis* as one of the probiotics capable of producing GABA as future food additives that can improve human health and reduce the prevalence of some diseases disorders such as diabetes.

## Conclusion

Postbiotics, such as Gamma-aminobutyric acid, were shown to promote human health and to be effective as food additives. Furthermore, camel milk was conventionally regarded as a traditional function food. As a result, the current study proposes to evaluate the effects of KLDS_1.0727_ and KLDS_1.0373_ strains on hyperglycemia and hyperlipidemia in *STZ*-induced C_57_BL/6 mice. Our findings indicate that GABA and camel milk can regulate blood glucose levels in mice without causing serious injury to the organs and can improve the overall composition of blood plasma. The study’s findings suggested that using camel milk to manage type 1 diabetes has extremely promising results due to the high concentration of insulin-like protein. However, further research is needed before camel milk may be used as an alternative bio-resource for some medications like oral diabetes therapy. Furthermore, we recommended a greater emphasis on the creation of dietary items enriched with postbiotics GABA, as well as innovative pharmabiotics applications.

## Data Availability Statement

The raw data supporting the conclusions of this article will be made available by the authors, without undue reservation.

## Ethics Statement

The *in vivo* experiment was carried out in accordance with the Northeast Agricultural University’s institutional animal care and use committee guidelines, as well as the China Ministry of Science and Technology Guide for the Care and Use of Laboratory Animals, under the authorized protocol number specialized pathogen-free rodent management (SRM)-06.

## Author Contributions

AA and HA-M performed conceptualization, methodology, writing review, and editing. AA, GA, EA, HAJ, and HA-M performed investigation, and formal analysis. XCM performed supervision. All authors contributed to the study’s conception and design. All authors contributed to the article and approved the submitted version.

## Funding

This research was funded by the National Key Research and Development Program of China under Grant No. 2017YED0400304.

## Conflict of Interest

The authors declare that the research was conducted in the absence of any commercial or financial relationships that could be construed as a potential conflict of interest.

## Publisher’s Note

All claims expressed in this article are solely those of the authors and do not necessarily represent those of their affiliated organizations, or those of the publisher, the editors and the reviewers. Any product that may be evaluated in this article, or claim that may be made by its manufacturer, is not guaranteed or endorsed by the publisher.

## Footnotes

^1^ https://www.ncbi.nlm.nih.gov/genome/browse/#!/prokaryotes/lactobacillus%20brevis
